# Long non-coding RNA DANCR promotes cervical cancer growth via activation of the Wnt/β-catenin signaling pathway

**DOI:** 10.1186/s12935-020-1139-9

**Published:** 2020-02-22

**Authors:** Wanjia Tian, Ningjing Lei, Ruixia Guo, Zhongfu Yuan, Lei Chang

**Affiliations:** 1 Department of Gynecology, The First Affiliated Hospital of Zhengzhou University, No. 1 Jianshe Road, Zhengzhou, 450000 Henan China; 2 School of Basic Medical Sciences, Zhengzhou University, Zhengzhou, China

**Keywords:** Long non-coding RNA, Cervical cancer, Proliferation, Wnt/β-catenin signaling pathway

## Abstract

**Background:**

Long non-coding RNAs (lncRNAs) are implicated in many pathophysiological processes, including cancers. In particular, lncRNA DANCR is regarded as a cancer-associated lncRNA exerting various regulatory mechanisms. However, the expressions, functions, and mechanisms of action of DANCR in cervical cancer are still unclear.

**Methods:**

The expressions of DANCR in cervical cancer tissues and cell lines were evaluated using qRT-PCR. Correlations between DANCR expression and clinicopathological features and prognosis were analyzed. The roles of DANCR in cervical cancer growth were evaluated by in vitro CCK-8 and EdU assay, and in vivo xenograft assay. The regulatory effects of DANCR on Wnt/β-catenin signaling pathway were evaluated using nuclear proteins extraction, western blot, and qRT-PCR.

**Results:**

DANCR is increased in cervical cancer tissues and cell lines. Increased expression of DANCR is associated with large tumor size, advanced FIGO stage, and poor overall survival of cervical cancer patients. Functional experiments showed that enhanced expression of DANCR promotes cervical cancer cell proliferation in vitro and xenograft growth in vivo. Conversely, DANCR knockdown inhibits cervical cancer cell proliferation in vitro and xenograft growth in vivo. Mechanistic investigation demonstrated that DANCR upregulates the expressions of FRAT1 and FRAT2 and activates the Wnt/β-catenin signaling pathway. Blocking the Wnt/β-catenin signaling pathway abolishes the pro-proliferative roles of DANCR overexpression and anti-proliferative roles of DANCR knockdown.

**Conclusions:**

Our findings suggest DANCR as an oncogenic lncRNA in cervical cancer through activating the Wnt/β-catenin signaling pathway, and imply that DANCR may be a promising prognostic biomarker and therapeutic target for cervical cancer.

## Background

Cervical cancer is the second most common cancer in female, next to breast cancer, accounting for approximately 527,000 new cancer cases and 265,000 cancer deaths each year worldwide [1]. With great development of diagnostic and therapeutic strategies, including cervical cancer early screening, surgical resection, chemotherapy, and radiotherapy, the 5-year survival rate of cervical cancer has reached to 60–70% [2]. However, considerable cervical cancer patients’ long-term survivals are dismal [3]. Therefore, it is urgent to investigate the molecular mechanisms underpinning the initiation and progression of cervical cancer and develop better therapeutic modalities for cervical cancer therapy.

Accumulating evidences revealed that many non-coding RNAs, including microRNAs (miRNAs) and long non-coding RNAs (lncRNAs) are frequently deregulated and have critical roles in multiple cancers [4–10]. These non-coding RNAs could be novel candidates for prognostic biomarkers and therapeutic targets in various cancers including cervical cancer [9–13]. LncRNAs are a class of novel non-coding transcripts with more than 200 nucleotides in length [14, 15]. Next-generation sequencing technologies have identified more than 58,000 lncRNAs among human transcriptome, while the number of protein-coding genes is only 21,000 [16]. A variety of lncRNAs are revealed to be deregulated and associated with patients’ prognosis in several cancers including cervical cancer [17–20]. Furthermore, many lncRNAs are shown to regulate various biological aspects of cancer cells, such as cell proliferation, apoptosis, cell cycle, migration, invasion, drug-resistance, and so on [21–23]. These lncRNAs may be implicated in many signaling pathways critical for cancers, and regulate various oncogenes and tumor suppressors in different cancers [24–27].

LncRNA differentiation antagonizing non-protein coding RNA (DANCR), previously termed as ANCR, was first revealed to suppress progenitor differentiation [28]. Subsequent studies demonstrated that DANCR is a promising cancer-associated lncRNA [29]. DANCR is shown to be upregulated in gastric cancer, lung cancer, glioma, colorectal cancer, retinoblastoma, osteosarcoma, oesophageal cancer, breast cancer, prostate cancer, and hepatocellular carcinoma [30–37]. In these different cancers, DANCR mainly functions as an oncogene via promoting cell proliferation, invasion, migration, and/or inhibiting cell apoptosis [30–37]. However, the mechanisms underpinning the functions of DANCR in different cancers are various. DANCR was reported to modulate PI3K-Akt pathway in osteosarcoma, β-catenin pathway in hepatocellular carcinoma, miR-634-RAB1A signaling pathway in glioma, androgen-AR signaling pathway in prostate cancer [31, 32, 38, 39]. In cervical cancer, however, the expressions, roles, and mechanisms of action of DANCR are still unclear.

In the present study, we determined DANCR expression in cervical cancer tissues and cell lines, analyzed the correlation between DANCR expression and cervical cancer patients’ clinicopathological features, including prognosis. Furthermore, gain-of-function and loss-of-function assays were performed to investigate the biological roles of DANCR in cervical cancer growth. Finally, using public available dataset, combined with experimental verification, we investigated the mechanisms underlying the biological effects of DANCR in cervical cancer. We demonstrated that DANCR, which is an oncogenic lncRNA in cervical cancer through activating the Wnt/β-catenin signaling pathway, might be a promising prognostic biomarker and therapeutic target for cervical cancer.

## Methods

### Patient tissue samples

A total of 82 pairs of cervical cancer and adjacent noncancerous cervix tissues were obtained from cervical cancer patients with informed written consent who underwent potentially curative surgery in the First Affiliated Hospital of Zhengzhou University (Zhengzhou, China) from 2014 to 2016. All tissue samples were diagnosed by histopathological examination. Freshly resected tissue specimens were immediately frozen in liquid nitrogen and stored at − 80 ℃ until use. The Medical Ethics Committee of the First Affiliated Hospital of Zhengzhou University reviewed and approved this program in accordance with Helsinki Declaration.

### Cell culture and treatment

The human normal cervical epithelial cell line HCerEpiC was purchased from ScienCell Research Laboratories (Carlsbad, CA, USA). The cervical cancer cell lines HeLa, SiHa, C-33A, and ME-180 were purchased from American Type Culture Collection (Rockville, MD, USA). HCerEpiC cells were grown in Cervical Epithelial Cell Growth Supplement (ScienCell, Carlsbad, CA, USA). HeLa, SiHa, and C-33A cells were grown in Eagle’s Minimum Essential Medium (Invitrogen Carlsbad, CA, USA). ME-180 cells were grown in McCoy’s 5A Medium (Sigma-Aldrich, St. Louis, MO, USA). All media were added with 10% fetal bovine serum (Gibco, Grand Island, NY, USA). Where indicated, the cervical cancer cells were treated with 5 μM ICG-001 (Selleck Chemicals, Houston, TX, USA). All cell lines were grown in a humidified incubator with 5% CO_2_ at 37 ℃.

### RNA extraction, reverse transcription, and quantitative real-time PCR (qRT-PCR)

Total RNA was extracted from indicated tissues and cells with TRIzol Regent (Invitrogen) following the protocol. After being treated with DNase I (Takara, Dalian, China) to remove genomic DNA, the isolated RNA was subjected to reverse transcription using a PrimeScript RT Reagent Kit (Takara) following the protocol. Next, the cDNA was subjected to quantitative Real-Time PCR (qRT-PCR) using SYBR Premix Ex Taq (Takara) and gene specific primers. Primer sequences are as follows: for DANCR, 5′-GCGCCACTATGTAGCGGGTT-3′ (sense) and 5′-TCAATGGCTTGTGCCTGTAGTT-3′ (antisense); for FRAT1, 5′-TGGAAGCGAGAGTAAAAAGC-3′ (sense) and 5′-GGTCACGCCAAATAAGGAG-3′ (antisense); for FRAT2, 5′-TACCTCACTTAGCCCTTGG-3′ (sense) and 5′-ATGCGTGTCGTTAGTTTTCA-3′ (antisense); for C-myc, 5′-GCTGCTTAGACGCTGGATTT-3′ (sense) and 5′-CTCCTCCTCGTCGCAGTAGA-3′ (antisense); for Cyclin D1, 5′-TTCCTGTCCTACTACCGC-3′ (sense) and 5′-CTCCTCCTCTTCCTCCTC-3′ (antisense); and for β-actin, 5′-GGGAAATCGTGCGTGACATTAAG-3′ (sense) and 5′-TGTGTTGGCGTACAGGTCTTTG-3′ (antisense). The quantification of RNA expression was calculated following the comparative Ct method. β-actin was used as endogenous control.

### Plasmids construction and transfection

DANCR expressing plasmid pcDNA3.1-DANCR was constructed as previously described [40]. Briefly, DANCR full-length sequence was PCR amplified using the PrimeSTAR HS DNA polymerase (Takara) and the primers 5′-CCCAAGCTTGCCCTTGCCCAGAGTCTTC-3′ (sense) and 5′-CGGGATCCGTCAGGCCAAGTAAGTTTATTAAC-3′ (antisense). Next, the PCR products were subcloned into the Hind III and BamH I sites of pcDNA3.1. DANCR knockdown plasmid was constructed as previously described [38]. Briefly, one pair of cDNA oligonucleotides specifically targeting DANCR was inserted into the shRNA expression plasmid pGPH1/Neo (GenePharma, Shanghai, China). The sequences of DANCR specific shRNA were: 5′-CACCAGCCAACTATCCCTTCAGTTACATTCAAGAGATGTAACTGAAGGGATAGTTGGCTTTTTTTG-3′ (sense) and 5′-GATCCAAAAAAAGCCAACTATCCCTTCAGTTACATCTCTTGAATGTAACTGAAGGGATAGTTGGCT-3′ (antisense). The sequences of control scrambled shRNA were: 5′-CACCGTTCTCCGAACGTGTCACGTTTCAAGAGAACGTGACACGTTCGGAGAATTTTTTG-3′ (sense) and 5′-GATCCAAAAAATTCTCCGAACGTGTCACGTTCTCTTGAAACGTGACACGTTCGGAGAAC-3′ (antisense). Plasmids transfection was carried out using Lipofectamine 3000 (Invitrogen) according to the manufacturer’s protocols.

### Stable cell lines construction

For construction of DANCR stably overexpressed and control HeLa cells, DANCR expressing plasmid pcDNA3.1-DANCR or control empty plasmid pcDNA3.1 was transfected into HeLa cells. After 48 h, the transfected HeLa cells were treated with neomycin for 4 weeks. To construct DANCR stably depleted and control C-33A cells, DANCR specific shRNA or control scrambled shRNA was transfected into C-33A cells. After 48 h, the transfected C-33A cells were treated with neomycin for 4 weeks. The efficiencies of overexpression and knockdown were determined by qRT-PCR.

### Cell proliferation assays

Cell proliferation was assessed by Cell Counting Kit-8 (CCK-8) and Ethynyl deoxyuridine (EdU) assays. For CCK-8 assay, DANCR stably overexpressed or depleted cervical cancer cells were resuspended and plated into 96-well plates at a density of 3000 cells per-well. After culture for indicated time, CCK-8 solution (Beyotime, Shanghai, China) was added into the wells. After 2 h of continued culture, absorbance values of optical density (OD) at 450 nm for each well were detected using automatic enzyme-linked immune detector. EdU assay was performed with the Cell-Light™ EdU Apollo^®^643 In Vitro Imaging Kit (RiboBio, Guangzhou, China) according to the instructions. Results were analyzed by Zeiss fluorescence photomicroscope (Carl Zeiss, Oberkochen, Germany) via randomly counting ten fields.

### Mouse xenograft model

1 × 10^6^ DANCR stably overexpressed or depleted cervical cancer cells were resuspended in 100 μl phosphate buffered saline containing 50% matrigel (Invitrogen) and then subcutaneously injected into the flanks of female BALB/c-nu mice of 6 weeks old. The mice were maintained in a sterile environment on a daily 12-h light/12-h dark cycle. Subcutaneous tumor volumes were measured every 3 days using caliper and calculated following the formula: tumor volumes = 0.5 × length × width^2^. At the twenty-first day after injection, the mice were sacrificed and subcutaneous tumors were resected and weighed. The Animal Ethics Committee of the First Affiliated Hospital of Zhengzhou University reviewed and approved this program. Ki67 immunohistochemical staining was carried out as we previously described with Ki67 primary antibody (Cell Signaling Technology, Boston, MA, USA) [41].

### Western blot

Nuclear proteins were extracted from DANCR stably overexpressed or depleted cervical cancer cells using CelLytic™ NuCLEAR™ Extraction Kit (Sigma-Aldrich). Total proteins were extracted from indicted cervical cancer cells using RIPA buffer (Beyotime). Next, nuclear proteins or total proteins were subjected to 12% sodium dodecyl sulfate–polyacrylamide gel electrophoresis (SDS-PAGE). After being transferred onto polyvinylidene fluoride (PVDF) microporous membrane (Millipore, Boston, MA, USA), the blots were incubated with primary antibodies against β-catenin (Abcam, Hong Kong, China), histone H3 (Abcam), FRAT1 (Abcam), FRAT2 (Sigma-Aldrich), C-myc (Cell Signaling Technology), Cyclin D1 (Cell Signaling Technology), or GAPDH (Cell Signaling Technology). Histone H3 was employed as a loading control for nuclear protein, and GAPDH was employed as a loading control for total protein. The blots were visualized with chemiluminescence.

### Statistical analysis

All statistical analyses were carried out using the GraphPad Prism 5.0 version (La Jolla, CA, USA). For comparisons, Wilcoxon signed-rank test, Pearson Chi square test, log-rank test, Student’s *t*-test, Mann–Whitney test, and Pearson correlation analysis were carried put as indicated. Results were considered as statistically significant when *p* < 0.05.

## Results

### Expression and clinical significances of DANCR in cervical cancer

**Fig. 1:**
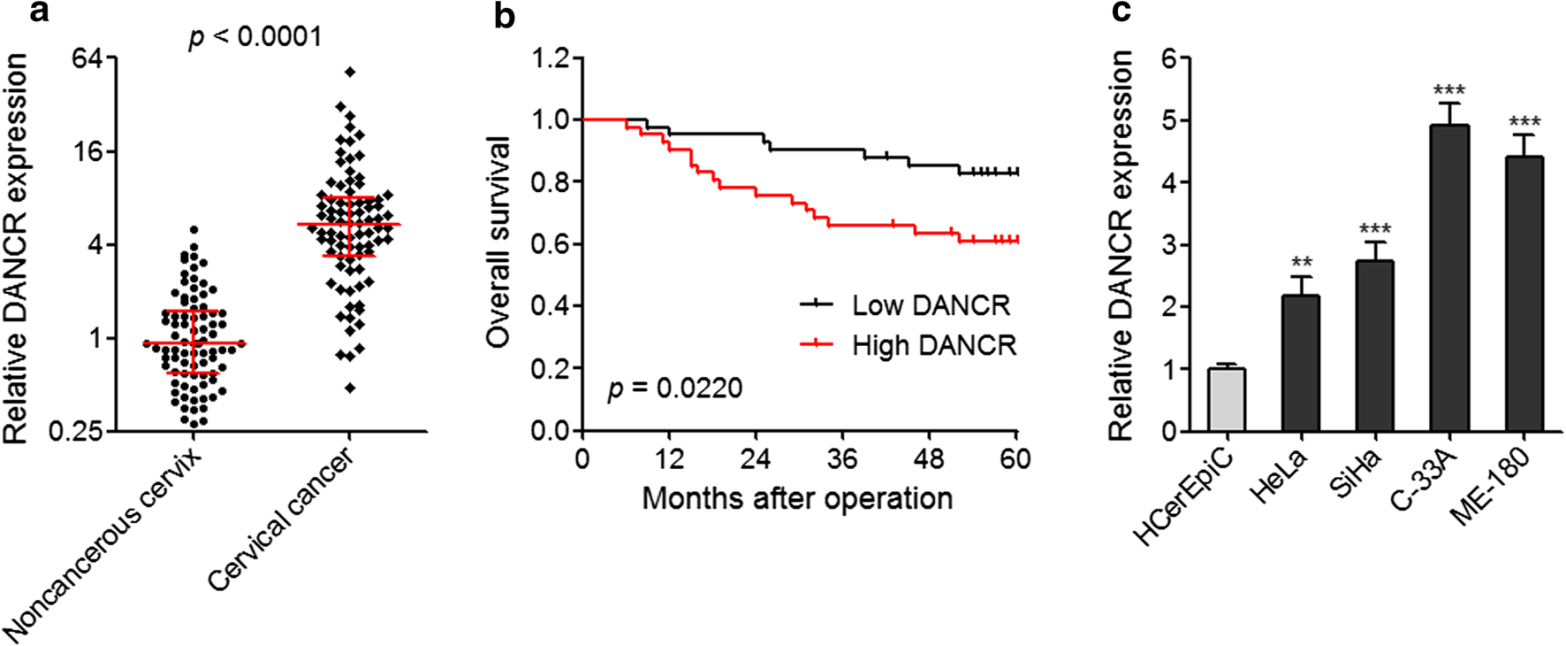
Expression pattern and clinical significances of DANCR in cervical cancer. **a** The expression of DANCR in 82 pairs of cervical cancer and adjacent noncancerous cervix tissues was measured by qRT-PCR. Results are displayed as median with interquartile range. *p* < 0.0001 by Wilcoxon signed-rank test. **b** Kaplan–Meier survival analysis of the association between DANCR expression and overall survival of cervical cancer patients (*n* = 82) *p* = 0.022 by log-rank test. DANCR median expression level was used as cut-off. **c** The expression of DANCR in human normal cervical epithelial cell line HCerEpiC and cervical cancer cell lines HeLa, SiHa, C-33A, and ME-180 was measured by qRT-PCR. Results are displayed as mean ± SD of *n* = 3 independent experiments. **p < 0.01, ***p < 0.001 compared with HCerEpiC group by one-way ANOVA

To determine the expression pattern of DANCR in cervical cancer, we firstly detected DANCR expression in 82 pairs of cervical cancer and adjacent noncancerous cervix tissues via qRT-PCR. Our results displayed that DANCR expression was markedly increased in cervical cancer tissues compared with adjacent noncancerous cervix tissues (Fig. 1a). To determine the association between DANCR expression and cervical cancer clinicopathological features, these 82 cervical cancer patients were divided into low and high DANCR expression groups based on DANCR median expression level. Pearson Chi square tests displayed that higher DANCR expression was positively associated with larger tumor size (*p* = 0.015) and advanced FIGO stage (*p* = 0.025) (Table 1). In addition, Kaplan–Meier survival analysis displayed that cervical cancer patients with higher DANCR expression had worse overall survival (*p* = 0.022) (Fig. 1b). Moreover, we measured DANCR expression in human normal cervical epithelial cell line HCerEpiC and cervical cancer cell lines HeLa, SiHa, C-33A, and ME-180 via qRT-PCR. As displayed in Fig. 1c, DANCR expression was obviously increased in cervical cancer cell lines compared with normal cervical epithelial cell line. Collectively, these data showed that DANCR is upregulated in cervical cancer tissues and cell lines, and increased expression of DANCR is positively correlated with larger tumor size, advanced FIGO stage, and worse prognosis.

**Table 1:** The association between DANCR expression and clinicopathological features in cervical cancer

Clinical features	DANCR		Chi square	*p* value
	Low	High		
All cases	41	41		
Age (year)			0.868	0.352
≥ 50	25	29		
< 50	16	12		
Tumor size (cm)			5.959	0.015
≥ 4	13	24		
< 4	28	17		
FIGO stage			5.025	0.025
I	29	19		
II	12	22		
Lymph node metastasis			2.121	0.145
Positive	9	15		
Negative	32	26		
Histology			0.069	0.794
Squamous	32	31		
Adenocarcinoma	9	10		

The median expression level of DANCR was used as cut-off

Pearson Chi square tests were used to analyze the association between DANCR expression and clinical features

### Upregulation of DANCR promotes cervical cancer growth in vitro and in vivo

**Fig. 2:**
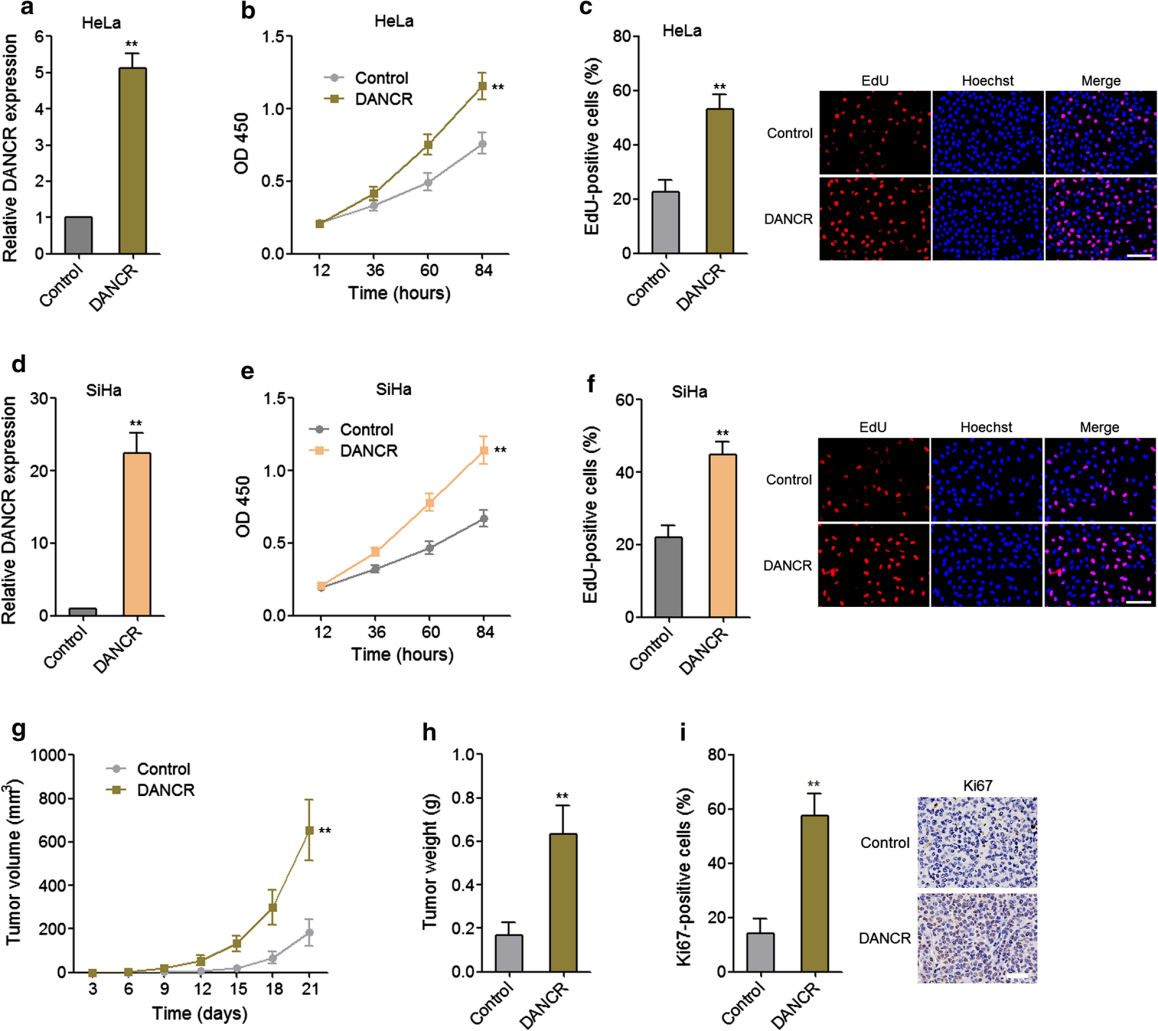
The effects of DANCR overexpression on cervical cancer growth. **a** The expression of DANCR in DANCR stably overexpressed (OV) and control HeLa cells was measured by qRT-PCR. **b** Cell proliferation of OV and control HeLa cells was analyzed by CCK-8 assays. OD 450 values at indicated time points were displayed to indicate cell proliferation. **c** Cell proliferation of OV and control HeLa cells was analyzed by EdU assays. Red colour represents EdU-positive and proliferative cells. Scale bars = 100 μm. **d** After transient transfection of DANCR expressing plasmid or control empty plasmid into SiHa cells, the expression of DANCR in the transfected cells was measured by qRT-PCR. **e** After transient transfection of DANCR expressing plasmid or control empty plasmid into SiHa cells, cell proliferation of the transfected cells was analyzed by CCK-8 assays. OD 450 values at indicated time points were displayed to indicate cell proliferation. **f** After transient transfection of DANCR expressing plasmid or control empty plasmid into SiHa cells, cell proliferation of the transfected cells was analyzed by EdU assays. Red colour represents EdU-positive and proliferative cells. Scale bars = 100 μm. For **a**–**f**, results are displayed as mean ± SD of *n* = 3 independent experiments. **p < 0.01 compared with Control group by Student’s *t*-test. **g** OV and control HeLa cells were subcutaneously injected into nude mice. Tumor volumes were measured every 3 days. **h** Subcutaneous tumor weights were detected at the twenty-first day after injection. **i** Proliferation marker Ki67 immunohistochemical staining in subcutaneous tumors formed by OV and control HeLa cells. Scale bars = 50 μm. For **g**–**i**, results are displayed as mean ± SD of *n* = 5 mice in each group. **p < 0.01 compared with Control group by Mann–Whitney test

To determine the functional relevance of DANCR in cervical cancer, we constructed DANCR stably overexpressed and control HeLa cells through transfecting DANCR expressing plasmid (pcDNA3.1-DANCR) or control empty plasmid (pcDNA3.1). The overexpression efficiency was detected via qRT-PCR (Fig. 2a). CCK-8 assays displayed that upregulation of DANCR promoted proliferation of HeLa cells (Fig. 2b). The pro-proliferative roles of upregulation of DANCR in HeLa cells were further confirmed by EdU assays (Fig. 2c). Furthermore, we transiently overexpressed DANCR in another cervical cancer cell line SiHa (Fig. 2d). CCK-8 assays displayed that upregulation of DANCR also promoted proliferation of SiHa cells (Fig. 2e). The pro-proliferative roles of upregulation of DANCR in SiHa cells were further confirmed by EdU assays (Fig. 2f). To further investigate the functions of DANCR in vivo, subcutaneous tumor model was employed via the subcutaneous injection of DANCR stably overexpressed and control HeLa cells. As displayed in Fig. 2g, h, upregulation of DANCR promoted subcutaneous tumor growth. Furthermore, proliferation marker Ki67 staining displayed that the subcutaneous tumor formed by DANCR overexpressed HeLa cells had much more Ki67 positive cells than that formed by control HeLa cells (Fig. 2i), which further supports the pro-proliferative roles of upregulation of DANCR in vivo. Collectively, these data showed that upregulation of DANCR promotes cervical cancer growth in vitro and in vivo.

### Knockdown of DANCR suppresses cervical cancer growth in vitro and in vivo

**Fig. 3:**
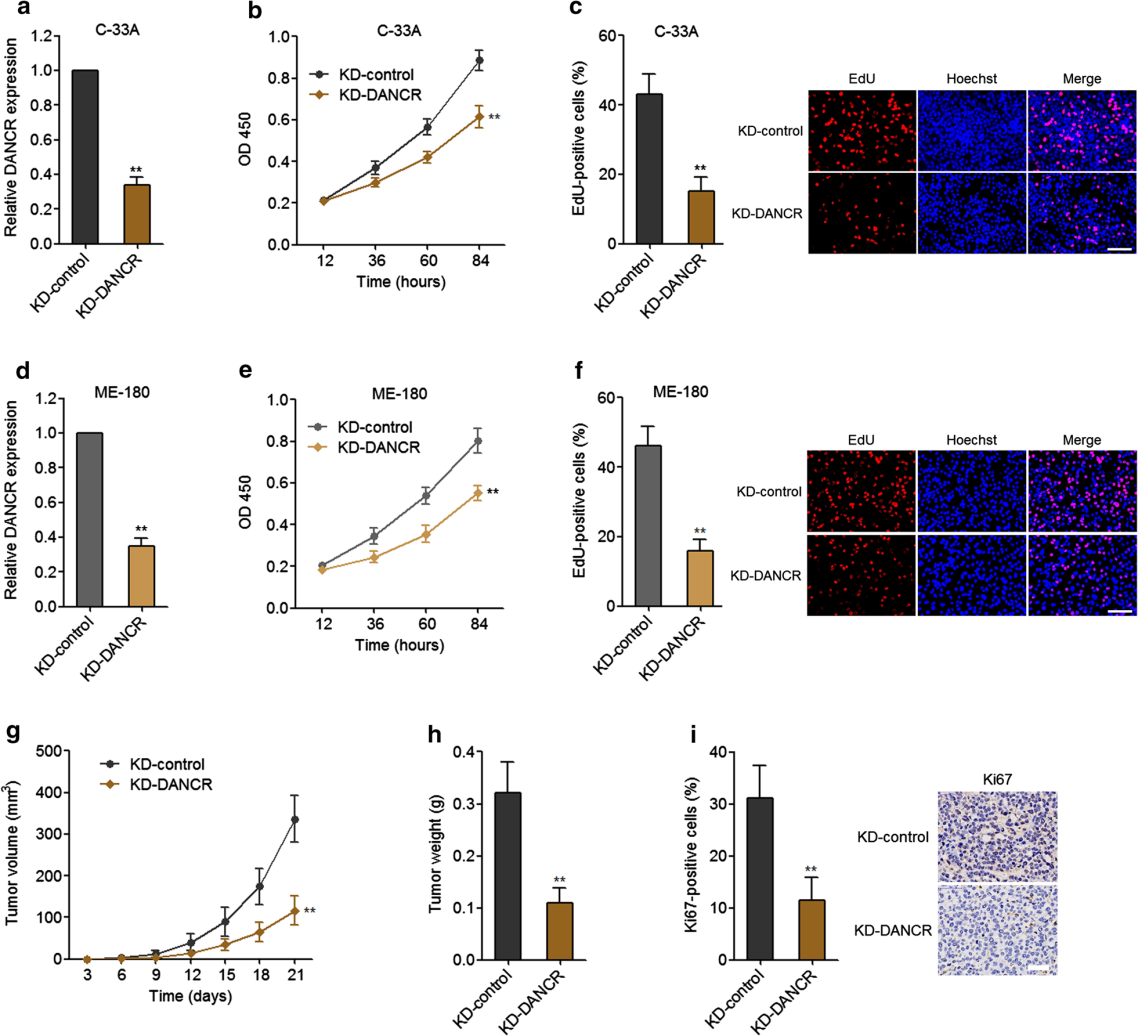
The effects of DANCR knockdown on cervical cancer growth. **a** The expression of DANCR in DANCR stably depleted (deleted) and control C-33A cells was measured by qRT-PCR. **b** Cell proliferation of deleted and control C-33A cells was analyzed by CCK-8 assays. OD 450 values at indicated time points were displayed to indicate cell proliferation. **c** Cell proliferation of deleted and control C-33A cells was analyzed by EdU assays. Red colour represents EdU-positive and proliferative cells. Scale bars = 100 μm. **d** After transient transfection of DANCR specific shRNA (KD-DANCR) or control scrambled shRNA (KD-control) into ME-180 cells, the expression of DANCR was measured by qRT-PCR. **e** After transient transfection, cell proliferation of KD-DANCR and KD-control ME-180 cells was analyzed by CCK-8 assays. OD 450 values at indicated time points were displayed to indicate cell proliferation. **f** After transient transfection of DANCR specific shRNA (KD-DANCR) or control scrambled shRNA (KD-control) into ME-180 cells, cell proliferation of the transfected cells was analyzed by EdU assays. Red colour represents EdU-positive and proliferative cells. Scale bars = 100 μm. For **a**–**f**, results are displayed as mean ± SD of *n* = 3 independent experiments. **p < 0.01 compared with KD-control group by Student’s *t*-test. **g** Deleted and control C-33A cells were subcutaneously injected into nude mice. Tumor volumes were measured every 3 days. **h** Subcutaneous tumor weights were detected at the twenty-first day after injection. **i** Proliferation marker Ki67 immunohistochemical staining in subcutaneous tumors formed by deleted and control C-33A cells. Scale bars = 50 μm. For **g**–**i**, results are displayed as mean ± SD of *n* = 5 mice in each group. **p < 0.01 compared with KD-control group by Mann–Whitney test

To further validate the functional relevance of DANCR in cervical cancer, we constructed DANCR stably depleted and control C-33A cells via transfecting DANCR specific shRNA (KD-DANCR) or control scrambled shRNA (KD-control). The knockdown efficiency was detected via qRT-PCR (Fig. 3a). CCK-8 assays displayed that knockdown of DANCR suppressed proliferation of C-33A cells (Fig. 3b). The anti-proliferative roles of knockdown of DANCR in C-33A cells were further confirmed by EdU assays (Fig. 3c). Furthermore, we transiently repressed DANCR in another cervical cancer cell line ME-180 (Fig. 3d). CCK-8 assays displayed that knockdown of DANCR also suppressed proliferation of ME-180 cells (Fig. 3e). The anti-proliferative roles of knockdown of DANCR in ME-180 cells were further confirmed by EdU assays (Fig. 3f). To investigate the roles of knockdown of DANCR in vivo, subcutaneous tumor model was employed via the subcutaneous injection of DANCR stably depleted and control C-33A cells. As displayed in Fig. 3g, h, knockdown of DANCR suppressed subcutaneous tumor growth. Furthermore, proliferation marker Ki67 staining displayed that the subcutaneous tumor formed by DANCR depleted C-33A cells had much less Ki67 positive cells than that formed by control C-33A cells (Fig. 3i), which further supports the anti-proliferative roles of knockdown of DANCR in vivo. Collectively, these data showed that knockdown of DANCR suppresses cervical cancer growth in vitro and in vivo.

### DANCR upregulates FRAT1 and FRAT2 expression

**Fig. 4:**
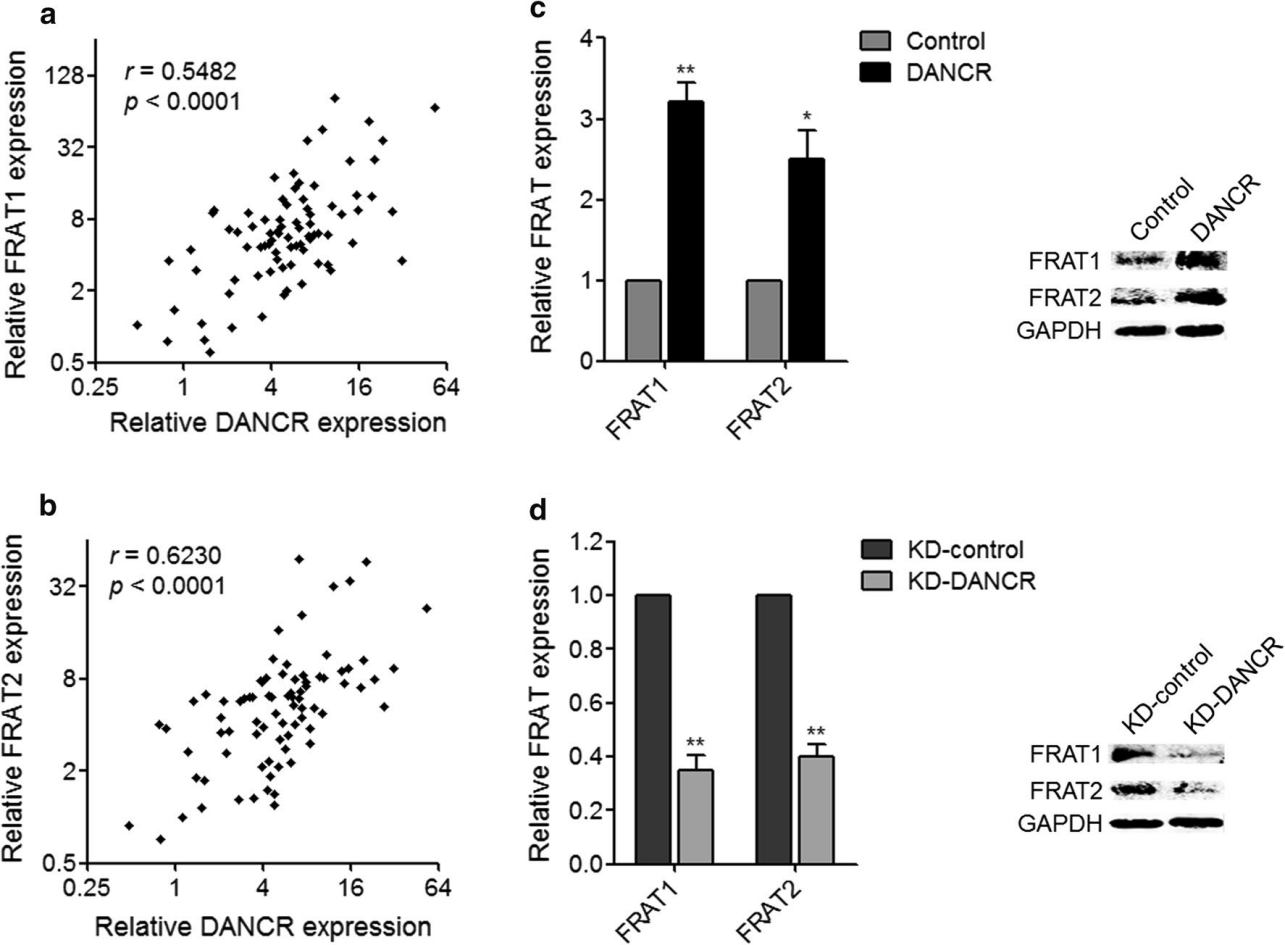
The effects of DANCR on FRAT1 and FRAT2. **a** The correlation between FRAT1 expression and DANCR expression in cervical cancer tissues was analyzed by Pearson correlation analysis. *n* = 82, *r* = 0.5482, *p* < 0.0001. **b** The correlation between FRAT2 expression and DANCR expression in cervical cancer tissues was analyzed by Pearson correlation analysis. *n* = 82, *r* = 0.623, *p* < 0.0001. **c** The mRNA and protein expression levels of FRAT1 and FRAT2 in DANCR stably overexpressed and control HeLa cells were measured by qRT-PCR and western blot. Results are displayed as mean ± SD of n = 3 independent experiments. *p < 0.05, **p < 0.01 compared with Control group by Student’s *t*-test. **d** The mRNA and protein expression levels of FRAT1 and FRAT2 in DANCR stably depleted and control C-33A cells were measured by qRT-PCR and western blot. Results are displayed as mean ± SD of n = 3 independent experiments. **p < 0.01 compared with KD-control group by Student’s *t*-test

To investigate the mechanisms underpinning the pro-proliferative roles of DANCR in cervical cancer, we searched the genes whose expression was correlated with that of DANCR in cervical cancer via analysing The Cancer Genome Atlas (TCGA) data using TANRIC (http://ibl.mdanderson.org/tanric/_design/basic/index.html). Among the genes positively associated with DANCR (Additional file 1: Table S1), we noted FRAT1 and FRAT2, which are positive regulator of the Wnt/β-catenin signaling pathway and have oncogenic roles in several cancers [42–45]. Therefore, we further analyzed the association between DANCR expression and FRAT1 and FRAT2 expression in 82 cervical cancer tissues used in Fig. 1. Pearson correlation analysis displayed that the expression of FRAT1 and FRAT2 were both positively associated with the expression of DANCR in cervical cancer tissues (Fig. 4a, b). We further measured the mRNA and protein expression levels of FRAT1 and FRAT2 in DANCR stably overexpressed and control HeLa cells, and DANCR stably depleted and control C-33A cells using qRT-PCR and western blot. Our results displayed that enhanced expression of DANCR upregulated the mRNA and protein expression levels of FRAT1 and FRAT2, and while knockdown of DANCR downregulated the mRNA and protein expression levels of FRAT1 and FRAT2 in cervical cancer cells (Fig. 4c, d).

### DANCR activates the Wnt/β-catenin signaling pathway

**Fig. 5:**
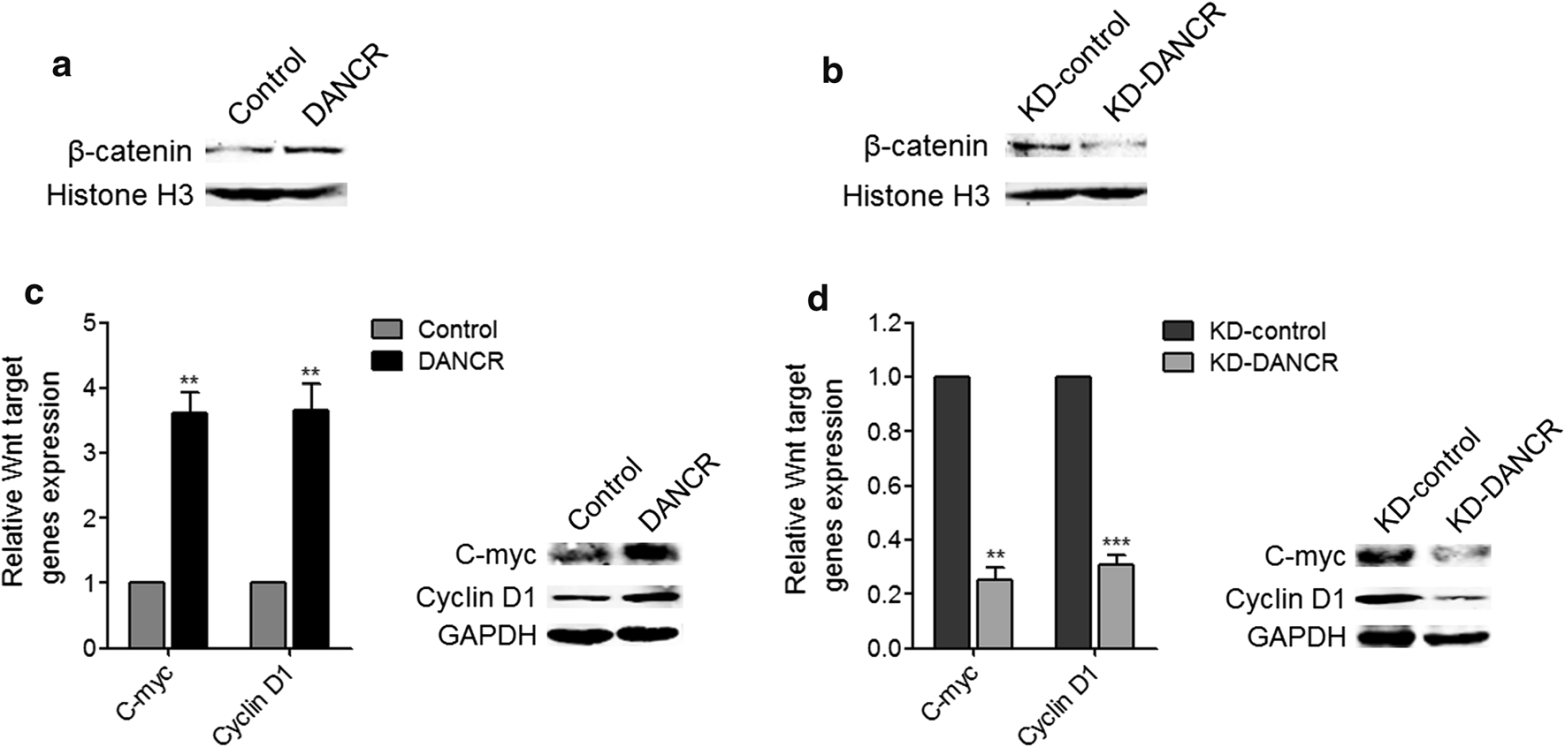
The effects of DANCR on the Wnt/β-catenin signaling pathway. **a** Nuclear β-catenin expression levels in DANCR stably overexpressed and control HeLa cells was measured by western blot. Results are displayed as representative image of n = 3 independent experiments. **b** Nuclear β-catenin expression levels in DANCR stably depleted and control C-33A cells was measured by western blot. Results are displayed as representative image of n = 3 independent experiments. **c** The mRNA and protein expression levels of C-myc and Cyclin D1 in DANCR stably overexpressed and control HeLa cells were measured by qRT-PCR and western blot. Results are displayed as mean ± SD of n = 3 independent experiments. **p < 0.01 compared with Control group by Student’s *t*-test. **d** The mRNA and protein expression levels of C-myc and Cyclin D1 in DANCR stably depleted and control C-33A cells were measured by qRT-PCR and western blot. Results are displayed as mean ± SD of n = 3 independent experiments. **p < 0.01, ***p < 0.001 compared with KD-control group by Student’s *t*-test

Next, we investigated the effects of DANCR on the Wnt/β-catenin signaling pathway in cervical cancer. Nuclear proteins from DANCR stably overexpressed and control HeLa cells, and DANCR stably depleted and control C-33A cells were extracted. Nuclear β-catenin levels were determined using western blot. The results displayed that enhanced expression of DANCR upregulated nuclear β-catenin levels, and while knockdown of DANCR downregulated nuclear β-catenin levels in cervical cancer cells (Fig. 5a, b). Furthermore, the mRNA and protein expression levels of Wnt target genes C-myc and Cyclin D1 in DANCR stably overexpressed and control HeLa cells, and DANCR stably depleted and control C-33A cells were measured using qRT-PCR and western blot. Our results displayed that enhanced expression of DANCR upregulated the mRNA and protein expression levels of C-myc and Cyclin D1, and while knockdown of DANCR downregulated the mRNA and protein expression levels of C-myc and Cyclin D1 in cervical cancer cells (Fig. 5c, d). Collectively, these data demonstrated that DANCR activates the Wnt/β-catenin signaling pathway.

### Wnt pathway inhibitor ICG-001 abolishes the roles of DANCR in cervical cancer growth

**Fig. 6:**
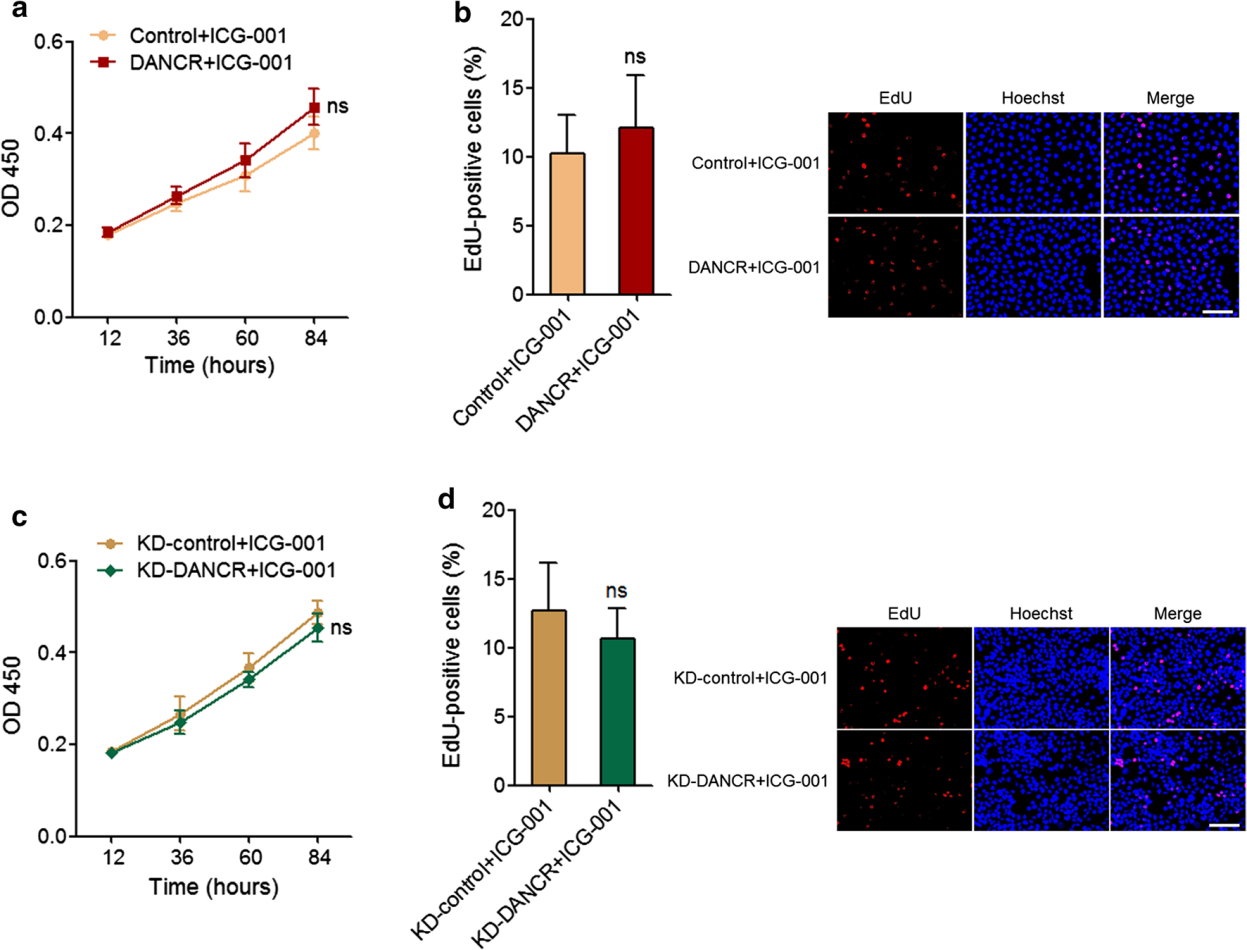
Wnt pathway inhibitor ICG-001 abolishes the roles of DANCR in cervical cancer growth. **a** DANCR stably overexpressed and control HeLa cells were treated with 5 μM ICG-001. Concurrently, cell proliferation was analyzed by CCK-8 assays. OD 450 values at indicated time points were displayed to indicate cell proliferation. **b** DANCR stably overexpressed and control HeLa cells were treated with 5 μM ICG-001. Concurrently, cell proliferation was analyzed by EdU assays. Red color represents EdU-positive and proliferative cells. Scale bars = 100 μm. For **a**, **b**, results are displayed as mean ± SD of *n* = 3 independent experiments. ns, not significant, compared with Control + ICG-001 group by Student’s *t*-test. **c** DANCR stably depleted and control C-33A cells were treated with 5 μM ICG-001. Concurrently, cell proliferation was analyzed by CCK-8 assays. OD 450 values at indicated time points were displayed to indicate cell proliferation. **d** DANCR stably depleted and control C-33A cells were treated with 5 μM ICG-001. Concurrently, cell proliferation was analyzed by EdU assays. Red color represents EdU-positive and proliferative cells. Scale bars = 100 μm. For **c**, **d**, results are displayed as mean ± SD of *n* = 3 independent experiments. ns, not significant, compared with KD-control + ICG-001 group by Student’s *t*-test

To investigate whether the activation of the Wnt/β-catenin signaling pathway mediates the pro-proliferative roles of DANCR in cervical cancer, we treated DANCR stably overexpressed and control HeLa cells, and DANCR stably depleted and control C-33A cells with the canonical Wnt/β-catenin pathway inhibitor ICG-001. CCK-8 and EdU assays displayed that treatment with ICG-001 abolished the pro-proliferative roles of DANCR overexpression (Fig. 6a, b). Consistently, treatment with ICG-001 also abolished the anti-proliferative roles of DANCR knockdown (Fig. 6c, d). These data suggested that Wnt/β-catenin signaling pathway is responsible for the roles of DANCR in cervical cancer cell proliferation.

## Discussion

Genomic and molecular features of cervical cancer are complex [46, 47]. Accumulating studies have identified various genomic mutations of ***PIK3CA***, ***EP300***, ***FBXW7***, ***PTEN***, ***HLA*-*A***, ***ARID1A*** and so on in different cervical cancer tissues [11, 46]. The aberrant expression of many mRNAs, including FGFR3, CTGF, TP63, IL36G, ADH7, SPINK5, and so on, have also been identified in cervical cancer [47]. Furthermore, the dysregulation of non-coding RNAs gradually attracts researchers’ attention, such as miR-205, miR-200a, miR-30a, lncRNA BCAR4, lncRNA HOTAIR, lncRNA MALAT1, lncRNA MEG3, and so on [46, 47]. Due to the huge amount of lncRNAs, the clinical significances of most of lncRNAs in cervical cancer are unclear.

In the present study, we focused on a lncRNA DANCR, which is located on chromosome 4q12. Although DANCR has been investigated in several cancers, and been regarded as a cancer-associated lncRNA [29], the functions and clinical significances of DNACR in cervical cancer are unclear. In this study, we identified DANCR is upregulated in cervical cancer tissues and cell lines compared with adjacent noncancerous cervix tissues and normal cervical epithelial cell line, respectively. High expression of DANCR is positively associated with large tumor size, advanced FIGO stage, and poor overall survival of cervical cancer patients. Functional experiments demonstrated that ectopic expression of DANCR promotes cervical cancer cell proliferation in vitro and cervical cancer xenograft growth in vivo. Conversely, DANCR knockdown inhibits cervical cancer cell proliferation in vitro and cervical cancer xenograft growth in vivo. Therefore, our data demonstrated that DANCR also functions as an oncogene in cervical cancer, further supporting DANCR as a cancer-associated lncRNA. Our findings also implied that DANCR might be a promising prognostic biomarker and therapeutic target for cervical cancer.

In this study, we identified a novel mechanism mediating the oncogenic roles of DANCR in cervical cancer, which is the activation of the Wnt/β-catenin signalling pathway via upregulation of FRAT1 and FRAT2. Both public available TCGA data and cervical cancer tissues we collected display that the expression of FRAT1 and FRAT2 are positively associated with the expression of DANCR in cervical cancer tissues, supporting the positive regulation of FRAT1 and FRAT2 by DANCR. FRAT1 and FRAT2 belong to the GSK-3-binding protein family, inhibit GSK-3-mediated β-catenin phosphorylation and degradation, promote nuclear translocation of β-catenin, and activate Wnt/β-catenin signaling pathway [44]. Indocyanine Green-001 (ICG-001) is an antagonist of β-catenin that specifically downregulates the expression of responsive genes of β-catenin [48]. Thus, we used ICG-001 to inhibit Wnt/β-catenin signaling pathway in the functional assays, which led to the abolishment of the pro-proliferation of cervical cancer cells caused by DANCR overexpression and the anti-proliferatory roles of DNACR knockdown in cervical cancer cells. The results of functional experiments suggest that the effects of DANCR on cervical cancer cells are dependent on the activation of Wnt/β-catenin signaling pathway. DANCR has previously been reported to activate the Wnt/β-catenin signaling pathway in hepatocellular carcinoma, gastric cancer, and glioma [32, 35, 48]. However, the detailed mechanisms underpinning the activation of the Wnt/β-catenin signaling pathway by DANCR in gastric cancer and glioma are unreported 9 [35, 49]. In hepatocellular carcinoma, Yuan et al. reported that DANCR directly bound to β-catenin mRNA and inhibited β-catenin mRNA degradation [32]. Several recent studies have reported the role of DANCR in cervical cancer associated with certain miRNAs [50, 51], however whether DANCR also affects the Wnt/β-catenin signalling pathway in cervical cancer has not been revealed. In this present study, we provide a novel insight that the activation of the Wnt/β-catenin signalling pathway by DANCR is associated with cervical cancer progression. In addition, we also identify that DANCR regulates the expression levels of FRAT1 and FRAT2, which are regulators of Wnt/β-catenin signalling pathway.

## Conclusions

In conclusion, our findings demonstrate that lncRNA DANCR is upregulated in cervical cancer, positively associated with tumor size and FIGO stage, and a prognostic indicator of poor survival of cervical cancer patients. DANCR promotes cervical cancer growth via activating FRAT1/FRAT2-Wnt/β-catenin signaling pathway. Our results imply that DANCR may be a promising prognostic biomarker to evaluate cervical cancer progression and useful therapeutic target for cervical cancer treatment.

## Supplementary information

**Additional file 1: Table S1.** The genes associated with DANCR in cervical cancer from TCGA data.

## Data Availability

All data generated or analysed during the present study are included in this published article.

## Abbreviations

lncRNA: Long non-coding ribonucleic acid
qRT-PCR: Quantitative real time polymerase chain reaction
CCK-8: Cell Counting Kit-8
EdU: Ethynyl deoxyuridine
FIGO: International federation of gynecology and obstetrics
OD: Optical density
SDS-PAGE: Sodium dodecyl sulfate–polyacrylamide gel electrophoresis
PVDF: Polyvinylidene fluoride
ICG-001: Indocyanine Green-001
FRAT: Frequently rearranged in advanced T cell lymphomas
