# Supplementary material for: Long non-coding RNA DANCR promotes cervical cancer growth via activation of the Wnt/β-catenin signaling pathway
Source: Cancer Cell Int. 2020 Feb 22;20:61. doi: 10.1186/s12935-020-1139-9 (PMC7036257; doi:10.1186/s12935-020-1139-9)
Supplement: Supplementary file 1 — Additional file 1: Table S1. The genes associated with DANCR in cervical cancer from TCGA data. [file 12935_2020_1139_MOESM1_ESM.docx]

**Additional file 1: Table S1.** The genes associated with DANCR in cervical cancer from TCGA data

| Cancer | Gene Symbol | Correlation | *p* value |
| --- | --- | --- | --- |
| TCGA-CESC | **FRAT2** | **0.598** | 0 |
| TCGA-CESC | C9orf140 | 0.5 | 2.75E-07 |
| TCGA-CESC | TPRN | 0.499 | 2.85E-07 |
| TCGA-CESC | SUPV3L1 | 0.494 | 3.96E-07 |
| TCGA-CESC | GCDH | 0.478 | 0.00000101 |
| TCGA-CESC | C10orf2 | 0.471 | 0.00000153 |
| TCGA-CESC | AKAP1 | 0.469 | 0.00000169 |
| TCGA-CESC | ACTR3B | 0.467 | 0.00000194 |
| TCGA-CESC | MAZ | 0.464 | 0.00000219 |
| TCGA-CESC | ACVR2B | 0.461 | 0.0000026 |
| TCGA-CESC | TMEM180 | 0.454 | 0.00000381 |
| TCGA-CESC | FARSA | 0.452 | 0.00000431 |
| TCGA-CESC | CABC1 | 0.45 | 0.00000491 |
| TCGA-CESC | IMPDH2 | 0.45 | 0.00000483 |
| TCGA-CESC | POU5F1B | 0.443 | 0.00000694 |
| TCGA-CESC | ABHD14B | 0.44 | 0.00000815 |
| TCGA-CESC | CLN3 | 0.44 | 0.00000832 |
| TCGA-CESC | **FRAT1** | **0.439** | 0.00000868 |
| TCGA-CESC | SLC25A23 | 0.439 | 0.00000872 |
| TCGA-CESC | ZNF589 | 0.435 | 0.0000103 |
| TCGA-CESC | ECHS1 | 0.433 | 0.0000115 |
| TCGA-CESC | PRDX2 | 0.431 | 0.0000129 |
| TCGA-CESC | CHERP | 0.43 | 0.0000138 |
| TCGA-CESC | ECSIT | 0.43 | 0.0000138 |
| TCGA-CESC | FAM53C | 0.426 | 0.0000167 |
| TCGA-CESC | FBXW9 | 0.426 | 0.0000164 |
| TCGA-CESC | MXI1 | 0.426 | 0.0000165 |
| TCGA-CESC | SFT2D3 | 0.424 | 0.0000179 |
| TCGA-CESC | ACN9 | 0.421 | 0.0000211 |
| TCGA-CESC | CHDH | 0.42 | 0.0000217 |
| TCGA-CESC | GOT1 | 0.42 | 0.0000216 |
| TCGA-CESC | NT5DC2 | 0.419 | 0.0000236 |
| TCGA-CESC | TEX264 | 0.418 | 0.0000241 |
| TCGA-CESC | MRPS16 | 0.417 | 0.0000249 |
| TCGA-CESC | C18orf55 | 0.416 | 0.0000265 |
| TCGA-CESC | DAZAP1 | 0.411 | 0.000034 |
| TCGA-CESC | KCNJ11 | 0.41 | 0.000036 |
| TCGA-CESC | ZNF485 | 0.41 | 0.0000362 |
| TCGA-CESC | MRPS25 | 0.409 | 0.000038 |
| TCGA-CESC | TMEM97 | 0.409 | 0.0000376 |
| TCGA-CESC | GNRHR2 | 0.408 | 0.0000396 |
| TCGA-CESC | TMEM161A | 0.408 | 0.0000383 |
| TCGA-CESC | ZFYVE27 | 0.408 | 0.0000393 |
| TCGA-CESC | MEPCE | 0.406 | 0.0000426 |
| TCGA-CESC | ASB13 | 0.405 | 0.0000461 |
| TCGA-CESC | SFXN4 | 0.405 | 0.0000451 |
| TCGA-CESC | BCCIP | 0.404 | 0.0000479 |
| TCGA-CESC | PSTK | 0.403 | 0.0000498 |
| TCGA-CESC | TUBGCP2 | 0.403 | 0.0000502 |
| TCGA-CESC | UROS | 0.403 | 0.0000487 |
| TCGA-CESC | C18orf56 | 0.402 | 0.000052 |
| TCGA-CESC | H2AFZ | 0.401 | 0.0000548 |
